# Injuries and Diseases of the Jaws

**Published:** 1869-04

**Authors:** 


### BIBLIOGRAPHICAL NOTICES.

*Injuries and Diseases of the Jaws.*—The Jacksonian Prize Essay of the Royal College of Surgeons of England, 1867. By Christopher Heath, F. R. C. S. London: John Churchhill & Sons. Philadelphia: Lindsay & Blakiston.

The first four Chapters of this valuable work are devoted to the subject of Fracture of the Jaws, containing, among others, Gunning's, Bean's, Hamilton's and Lonsdale's apparatus, with full notes of interesting cases. Chap. V. is devoted to Gunshot Injuries of the Jaws, with accounts of remarkable recoveries from most frightful wounds of the face, including the maxillary bones. Chap. VI. treats of Dislocation of the Jaw, including Rare Forms of Dislocation, Congenital Dislocation, &c. Chap. VIII. treats of Necrosis. Chap. X. of Hyperostosis. Chap. XI. of Cystic Diseases of the Antrum. Chap. XII. of Cysts of Teeth. Chap. XIII. of Cysts in the Lower Jaw. Chap. XIV. of Tumours Connected with the Teeth. Chap. XV. treats of Diseases of the Gums, including Hypertrophy, Polypus, Vascular Growths, Epulis, &c. Chap. XVI of Tumours of the Hard Palate. Chap. XVII. of Growths within the Antrum.

Chap. XVIII. to Chap. XXVI. inclusive, treats of Tumours of the Upper and Lower Jaws, including all the varieties to which these parts are liable. Chap. XXVII. of Closure of the Jaws, and Chap. XXVIII. of Deformities of the Jaws, with some very interesting accounts of severe cases, including one very similar to Hullihen's celebrated case. The information to be derived from this work will prove of great service to the dentist in the practice of his profession, and it will form a valuable addition to his library.

The volume is embellished by 154 well executed illustrations on wood, and contains 416 pages.

